# 

**Published:** 1891-01-24

**Authors:** 


PRICE ONE PENNY.

No. 226, Vol. IX.—January 24th, 1891.

[Registered at the General Post Office as a Newspaper for transmission in the United Kingdom and Abroad.]

WITH EXTRA NURSING SUPPLEMENT.
Per Annum, 6s. 6d., post free.
Monthly Parts, 6d.; post free 8d.
Ditto per Annum, 8s., post free.

### THE HOSPITAL

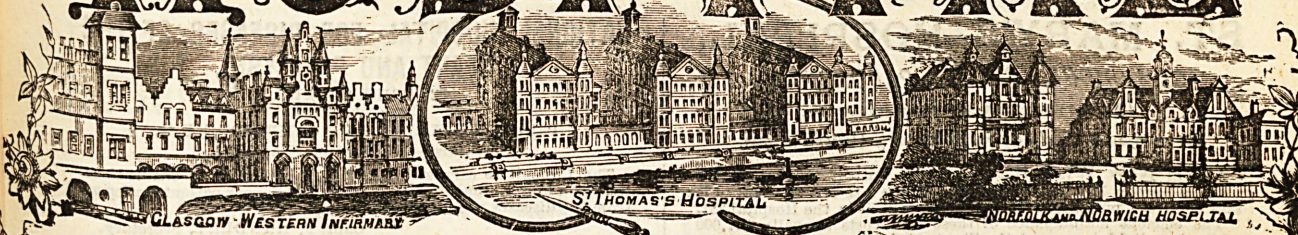

A WEEKLY JOURNAL OF

### Science, Medicine, Nursing and Philanthropy.

SATURDAY, JANUARY 24th, 1891.

**Virchow and Koch** . . . . . . . . . 259

**Passing Topics.**— 260

**The Doctor's Armchair.**—Lupus and Tuberculosis . . 261

**The Microscope in Medicine.** By FRANK J. WETHERED, M.D.—II. The Use of the Microscope . . . . . 262

**Everybody's Page** . . . . . . . . 263

**Notes and News** . . . . . . . . . 264

**General Charities** . . . . . . . . 265

**Scraps and Gleanings** . . . . . . . . 265

**Annotations.**—Doubtful Cases—A French Cure for Consumption—"We've Got no Work to Do" . . . . . 266

**Recreation in Relation to the Health of the People** 270

**The Practitioner's Mirror and Retrospect:**—

MEDICINE.—Treatment of Diphtheria—Spontaneous Expulsion of a Foreign Body from the Bronchi—Pilocarpine in Skin Diseases . . . . . . . . . . 267

SURGERY.—The Surgical Treatment of Encephalocele . . 267

TOXICOLOGY.—Pennyroyal—Strychnine Poisoning . . 268

THERAPEUTICS.—Bromethyl—Lanolin and Vaseline . . 268

NEW DRUGS, APPLIANCES, AND THINGS MEDICAL.—Aërated Waters for Hospitals—Pills—"Warrington" Chloroform—Robin's Ready-made Linseed Poultices . . . . . 269

**The Student of Medicine.**—Appointments—The Students' Bookshelf—Pass Lists.

### EXTRA SUPPLEMENT—THE NURSING MIRROR.

**En Passant.**—Murray Royal Asylum—The Golden Apple—Atcham Again—Liverpool Northern Hospital—Mrs. Ernest Hart—An Institute for Aylesbury—Short Items—Liverpool Jubilee Institute . . . . . . . lxxxix

**The Nurses' Co-operation** . . . . . . . xc

**Nursing Medals and Certificates**—The Butterworth Medal . . . . . . . . . . . xci

**Presentations** . . . . . . . . . . . xci

**For Reading to the Sick.**—Gratitude . . . . . xci

**Medical Applications of Electricity** . . . . . xcii

**Keeping Christmas** . . . . . . . . . xcii

**Everybody's Opinion.**—Nurses and Infection—The Pension Fund—Help Wanted—"In Darkest England." xciii

**Notes and Queries** . . . . . . . . . xciv

**Off Duty.**—A Winter Holiday. II.—Across the Channel . xciv

**Amusements and Relaxation** . . . . . . . xciv

